# Protracted lung repair after mild breakthrough Omicron SARS-CoV-2 infection: the storm beneath the calm

**DOI:** 10.1183/23120541.00350-2026

**Published:** 2026-10-26

**Authors:** Shreya M. Kanth, Honghui Wang, Xin Tian, Julio A. Huapaya, Salina Gairhe, Cumhur Y. Demirkale, Maura J. O'Neill, Thorkell Andresson, Junfeng Ma, Chunyan Hou, David Regenold, Anthony F. Suffredini

**Affiliations:** 1 Critical Care Medicine and Pulmonary Branch, National Heart, Lung, and Blood Institute, National Institutes of Health, Bethesda, MD, USA; 2 Critical Care Medicine Department, Clinical Center, National Institutes of Health, Bethesda, MD, USA; 3 Office of Biostatistics Research, National Heart, Lung, and Blood Institute, National Institutes of Health, Bethesda, MD, USA; 4 Protein and Metabolite Characterization Core, Cancer Research Technology Program, Frederick National Laboratory for Cancer Research, Frederick, MD, USA; 5 Mass Spectrometry and Analytical Pharmacology Shared Resource, Lombardi Comprehensive Cancer Center, Georgetown University Medical Center, Georgetown University, Washington, DC, USA; 6 Laboratory of Immunoregulation, National Institute of Allergy and Infectious Diseases, National Institutes of Health, Bethesda, MD, USA

## Abstract

Unlike the early COVID-19 pandemic, most patients with breakthrough infections from the Omicron variant of SARS-CoV-2 experience mild, short-lived symptoms with a rapid return to clinical baseline [1]. Despite the decrease in mortality after infection with the Omicron variant, there remains an increase in longstanding dysregulation of coagulation, immunometabolism and long COVID symptoms compared to uninfected persons [2–7]. Furthermore, there is limited information regarding the local pulmonary environment and reparative responses in Omicron-infected individuals.

## Abstract 2

**Proteomic analysis of serial bronchoalveolar lavage from 24 patients recovering from mild, self-limited Omicron-variant SARS-CoV-2 infection revealed a protracted pulmonary host repair response at a median of 281 days after symptom onset** https://bit.ly/4uk5bwC

*To the Editor*:

Unlike the early COVID-19 pandemic, most patients with breakthrough infections from the Omicron variant of SARS-CoV-2 experience mild, short-lived symptoms with a rapid return to clinical baseline [1]. Despite the decrease in mortality after infection with the Omicron variant, there remains an increase in longstanding dysregulation of coagulation, immunometabolism and long COVID symptoms compared to uninfected persons [2–7]. Furthermore, there is limited information regarding the local pulmonary environment and reparative responses in Omicron-infected individuals. This knowledge gap is due in part to limited sampling of the lower airways outside of a research indication. Longitudinal evaluation of the pulmonary host response after mild breakthrough infections is essential to understand the link between other acute viral pneumonias and the development of chronic pulmonary disorders.

We recently described the unexpected findings of prolonged lung reparative processes at 9 months after recovery from “pre-Omicron” SARS-CoV-2 infection [8]. Patients were enrolled in a natural history protocol (www.clinicaltrials.gov identifier number NCT04401449) during which bronchoscopy with bronchoalveolar lavage (BAL) was collected serially during acute through convalescent phases after infection. All participants provided informed consent in written or electronic forms. The study was approved by the National Institutes of Health institutional review board. High-density multiplatform proteomics were performed on the BAL, and revealed unanticipated evidence of ongoing activation of cellular repair processes and depression of the renin–kallikrein–kinin, coagulation and complement systems. Notably, these persistent subclinical biological processes occurred in the lung despite complete clinical resolution in this cohort. We thus investigated whether the lung proteome is similarly altered in persons with mild breakthrough SARS-CoV-2 infections with no residual cardiopulmonary abnormalities during the Omicron-predominant period.

Sequential BAL was collected from 24 minimally symptomatic patients (64 BAL samples) during the acute phase (n=24; median 28 days, interquartile range (IQR) 25–37 days), recovery phase (n=20; median 84 days, IQR 78–88 days), and convalescent phase (n=20; median 281 days, IQR 261–318 days) after initial symptom onset from COVID-19 breakthrough infection (figure 1a). Patients included in this study were diagnosed with COVID-19 breakthrough infections between January 2022 and March 2023, when the Omicron variant accounted for >99% of all SARS-CoV-2 infections in the Washington, DC, USA, metropolitan area [9]. This cohort had a median age of 44 years (IQR 37–52 years), with 16 (67%) females and a varied distribution of races: 12 (50%) White, eight (33%) Latino, two (8%) Black and two (8%) Asian. All patients had been fully vaccinated prior to their breakthrough infection, with eight (33%) most recently vaccinated with the mRNA-1283 (Moderna) and 16 (67%) with BNT162b2 (Pfizer-BioNTech) mRNA vaccines. None were hospitalised, and all had a mild disease course and recovered in the ambulatory setting. During the convalescent phase, two patients had persistent neuromuscular symptoms, consisting of mild exertional fatigue and “brain fog”, but none had residual cardiopulmonary symptoms. No significant parenchymal abnormalities were noted on chest computed tomography: two patients had mild dependent ground-glass abnormalities, one patient had a few bilateral lower-lobe thin-walled cysts and one patient had mild centrilobular emphysema.

**FIGURE 1:**
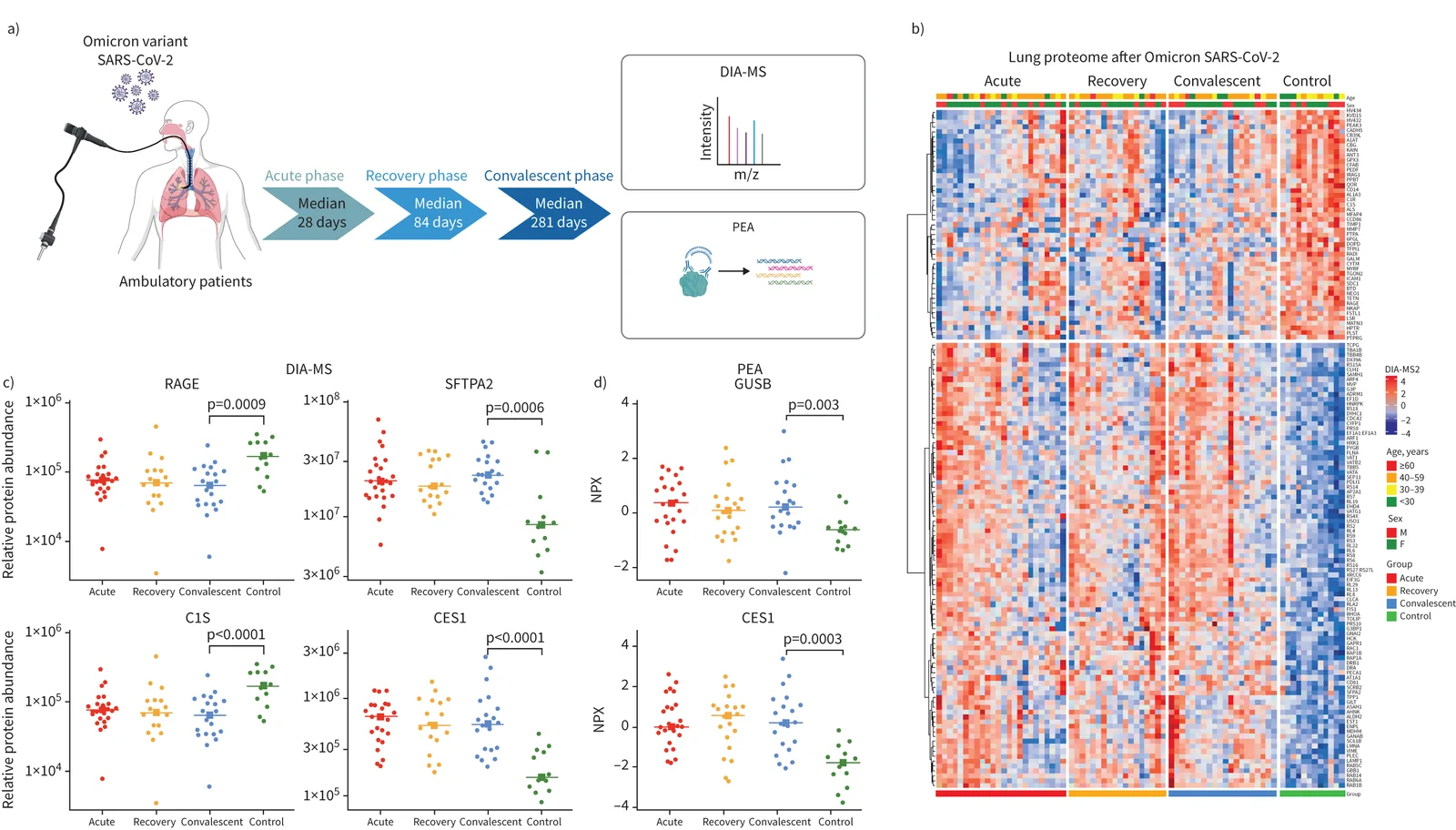
Ongoing cellular repair months after clinical recovery from Omicron-variant SARS-CoV-2 infection. a) Outline of protocol. Sequential bronchoalveolar lavage (BAL) sampling was performed across three phases over the course of a year. b) Heatmap of sequential expression of 138 proteins with differential expression in both acute and convalescent phases compared to control (false-discovery rate <5%, tandem mass spectrometry (MS2) spectra) by data-independent acquisition mass spectrometry (DIA-MS). c) Bee swarm plots of representative host response and lung repair proteins (receptor for advanced glycation end-products (RAGE), surfactant protein A2 (SFTPA2), complement component 1s (C1S) and carboxylesterase 1 (CES1)) at each phase compared to healthy control BAL by DIA-MS. d) Bee swarm plots of representative host response and lung repair proteins (β-glucuronidase (GUSB) and CES1) at each phase compared to healthy control BAL by proximal extension assay (PEA). NPX: normalised protein expression.

High-density proteomics was performed using a proximal extension assay (PEA) (Olink Explore 384 Cardiometabolic I and Inflammation I), which targeted 729 unique biomarkers, and data-independent acquisition mass spectrometry (DIA-MS), which identified 2820 proteins from all queried BAL samples. Total protein concentration and normalisation was performed across BAL samples for the DIA-MS analysis. Datasets from each platform were analysed independently. For PEA, all samples were processed on a single plate and processed at the same time. For DIA-MS, data acquisition was completed within 2 weeks, on the same machine, without interruption. Each sample was spiked with indexed retention time standard peptide to monitor the liquid chromatography/mass spectrometry performance during the 2-week data acquisition. The BAL from each phase was compared to control BAL (n=12; median age 33 years, IQR 27–39 years; 58% female) from uninfected healthy subjects obtained prior to the COVID-19 pandemic, from 2017–2019 (www.clinicaltrials.gov identifier number NCT02392442). Cellular composition analysis was not included in this study.

The proteomic signature of each COVID-19 phase was compared to control in both platforms using a fit ANCOVA model and ANOVA for PEA and DIA-MS, respectively. Paired longitudinal within-subject analysis to compare the acute, recovery and convalescent phases was performed. All comparisons were adjusted for age, race and sex. PEA results were also adjusted for the first principal component (PC1) as a latent variable to account for any unobserved confounding effects [10]. For DIA-MS, some proteins may not be detected in a single run within complex biological samples, leading to “missing values” [11]. Proteins missing in >50% of replicates within each comparator group were excluded and missing values from included proteins were imputed using the minimum value divided by 2, after combining the MS1 and MS2 spectra. Two recovery phase samples were excluded from the DIA-MS analysis due to technical processing errors. Biological pathway analysis was performed using Ingenuity Pathway Analysis (QIAGEN) and Metascape [12]. Differentially expressed protein (DEP) cutoffs included a false-discovery rate ≤5% and an absolute fold change ≥1.5.

Despite a mild clinical illness in all patients, comparison of acute phase *versus* healthy control BAL identified seven overexpressed DEPs by PEA (CES1, GZMA, GUSB, DIABLO, CTSZ, GGH and ATP5IF1) and 303 DEPs by DIA-MS (156 increased and 147 decreased, data available from https://tianxt.shinyapps.io/OmicronBAL/). Increased expression of BAL carboxylesterase 1 (CES1) overlapped between both proteomic platforms and was also found to be elevated in the pre-Omicron cohort [8]. Pathway analysis showed upregulation of neutrophil degranulation, cytokine signalling, extracellular matrix organisation, cellular apoptosis, integrated stress response signalling, and cellular and metabolic homeostasis pathways. Of note, there was concurrent compensatory downregulation of acute phase response signalling, complement cascade, cholesterol and lipid metabolism, and platelet activation and coagulation pathways. Notably, the convalescent phase BAL in the same cohort revealed five DEPs (CES1, B4GALT1, CA4, GUSB and SDC4) by PEA and 319 DEPs (157 increased and 162 decreased) by DIA-MS when compared to healthy controls (figure 1b–d). Pathway analysis showed a continued increase in the cellular response to stress and expression of metabolic repair pathways, and a sustained decrease in complement and coagulation cascades compared to healthy controls ∼9 months after symptom onset, similar to our prior study [8]. Sensitivity analyses using various imputation and absolute fold-change cutoffs showed consistent findings. No significant differences in protein expression were found when comparing acute to recovery and recovery to convalescent phases, suggesting that the host repair processes are initiated early after infection and are sustained well-beyond overt clinical improvement.

These findings highlight that in even in the setting of mild, self-limited respiratory viral infection, ongoing host repair processes are still present in the absence of residual symptoms and radiographic abnormalities. The protracted augmented expression of CES1 and β-glucuronidase (GUSB), for example, which are associated with pulmonary endothelial and gut endobiotic homeostasis, suggests an extended period to restore cellular health and return to biological baseline [13, 14]. Inhibition of proteins that propagate immune cell activation and tissue injury, such as complement component 1s (C1S) and receptor for advanced glycation end-products (RAGE), supports this hypothesis [15]. Our study was subject to certain inherent limitations, namely a small sample size, variability in time before presentation, and the absence of a comparative vaccinated and never-infected control group. Further exploration of the presence and consequences of longstanding host responses in other community-acquired viral pneumonias is necessary, particularly as drivers of chronic diseases such as reactive airways disease, progressive lung fibrosis or malignancies.
